# The IS*Vsa3*-ORF2-*abh*-*tet*(X4) circular intermediate-mediated transmission of tigecycline resistance in *Escherichia coli* isolates from duck farms

**DOI:** 10.3389/fcimb.2024.1444031

**Published:** 2024-08-30

**Authors:** Chao Jiang, Jie Yang, Gang Xiao, Ning Xiao, Jie Hu, Yi Yang, Zhiliang Sun, Yujuan Li

**Affiliations:** ^1^ College of Basic Medical Science, Xiangnan University, Chenzhou, Hunan, China; ^2^ Technology Research and Development Center of Chenzhou, Xiangnan University, Chenzhou, Hunan, China; ^3^ College of Veterinary Medicine, Hunan Agricultural University, Changsha, Hunan, China; ^4^ Hunan Engineering Technology Research Center of Veterinary Drugs, Hunan Agricultural University, Changsha, Hunan, China

**Keywords:** antimicrobial drug resistance, tigecycline, *tet*(X4), *Escherichia coli*, duck

## Abstract

Tigecycline is a last-resort drug used to treat serious infections caused by multidrug-resistant bacteria. *tet*(X4) is a recently discovered plasmid-mediated tigecycline resistance gene that confers high-level resistance to tigecycline and other tetracyclines. Since the first discovery of *tet*(X4) in 2019, it has spread rapidly worldwide, and as a consequence, tigecycline has become increasingly ineffective in the clinical treatment of multidrug-resistant infections. In this study, we identified and analyzed *tet*(X4)-positive *Escherichia coli* isolates from duck farms in Hunan Province, China. In total, 976 samples were collected from nine duck farms. Antimicrobial susceptibility testing and whole-genome sequencing (WGS) were performed to establish the phenotypes and genotypes of *tet*(X4)-positive isolates. In addition, the genomic characteristics and transferability of *tet*(X4) were determined based on bioinformatics analysis and conjugation. We accordingly detected an *E. coli* strain harboring *tet*(X4) and seven other resistance genes in duck feces. Multi-locus sequence typing analysis revealed that this isolate belonged to a new clone, and subsequent genetic analysis indicated that *tet*(X4) was carried in a 4608-bp circular intermediate, flanked by IS*Vsa3*-ORF2-*abh* elements. Moreover, it exhibited transferability to *E. coli* C600 with a frequency of 10^-5^. The detection of *tet*(X4)-harboring *E, coli* strains on duck farms enhances our understanding of tigecycline resistance dynamics. The transferable nature of the circular intermediate of *tet*(X4) contributing to the spread of tigecycline resistance genes poses a substantial threat to healthcare. Consequently, vigilant monitoring and proactive measures are necessary to prevent their spread.

## 1 Introduction

Antimicrobial resistance (AMR) has been recognized by the World Health Organization (WHO) as a major threat to global public health (O’Neill, 2016; Liu et al., 2024). Tigecycline is a glycylcycline antibiotic with an expanded spectrum of activity that was developed to address the global threat of emerging antibiotic resistance (Yahav et al., 2011). It is considered a last-resort treatment option for severe infections caused by multidrug-resistant bacteria (MDR) (Jenner et al., 2013), including *Enterobacteriaceae* strains resistant to carbapenem (CRE), *Staphylococcus aureus* resistant to methicillin (MRSA), and vancomycin-resistant *Enterococcus* (VRE) (Tasina et al., 2011; Sheu et al., 2019; Chen et al., 2020; Wu et al., 2022; Yaghoubi et al., 2022). However, the emergence of resistance genes from the *tet*(X) family [specifically *tet*(X4)] confers high resistance against tigecycline, posing significant challenges for successful treatment (Sun et al., 2019; Xu et al., 2022). Positive *tet*(X4) strains were first reported in porcine isolates of *Escherichia coli* in 2019. These strains can confer high-level resistance to all tetracycline antibiotics, including fourth-generation tetracycline drugs (eravacycline and omadacycline) recently approved by the United States Food and Drug Administration (FDA) (He et al., 2019). To date, *tet*(X4) genes have mainly been identified in ColE2-like, IncA/C2, IncX1, IncQ, IncFII, IncHI1, and other types of plasmids ranging in size from 9 to 330 kb with different replicons, which substantially enhances *tet*(X4) transferability (Ding et al., 2020; Aminov, 2021; Yu et al., 2021; Li et al., 2023). *tet*(X4) is present worldwide within a range of bacterial species that have been isolated from diverse ecological niches, including human and animal feces and the environment, among which, *Acinetobacter* spp., *Enterobacteriaceae*, and *E. coli* have been identified as the predominant reservoirs of *tet*(X4) (Zhang et al., 2022).

Although in recent years, the epidemiology and transmission of *tet*(X4) have been extensively reported, most of this research has been limited to pig farms and related production lines (Wang et al., 2022; Wu et al., 2022). Thus, conducting large-scale epidemiological and functional studies is essential to gain a better understanding of the prevalence and dissemination of *tet*(X4) in poultry. To this end, in the present study, we collected 976 samples from duck farms in Hunan Province, China, to investigate the epidemic and transmission characteristics of bacterial strains positive for *tet*(X4). Herein, we report the isolation of *E. coli* positive for *tet*(X4) from duck farms.

## 2 Methods

### 2.1 Collection of samples and bacterial isolation

In July 2022, a total of 976 non-duplicate samples were collected from nine duck farms in Hunan province, China, comprising 677 duck feces and 299 environmental-related samples. All samples were collected using sterile swabs and suspended in centrifuge tubes containing 1.5 mL of Luria–Bertani (LB) broth. Having immediately placed the samples in ice boxes, they were transported to the laboratory, wherein they cultured overnight at 37°C in a chromogenic medium suitable for urine cultures (Comagal, Shanghai, China) containing 4 µg/mL tigecycline. Colonies of different colors that subsequently developed were subjected to polymerase chain reaction (PCR) analysis to establish whether the selected isolates contained *tet*(X) resistance genes. Tet(X)-F/*tet*(X)-R primers (F: 5′-CCGTTGGACTGACTATGGC-3′, R: 5′-TCAACTTGCGTGTCGGTAA-3′) were used for Sanger sequencing (Sun et al., 2020). Subsequently, bacteria were identified at the species level based on 16S rRNA sequencing and comparison of sequencing results with the sequences of GenBank database reference strains (Gimenez-Miranda et al., 2023).

### 2.2 Antimicrobial susceptibility testing

The antimicrobial sensitivity of the *tet*(X4)-positive isolates to nine antibiotics (tigecycline, chloramphenicol, nalidixic acid, florfenicol, trimethoprim-sulfamethoxazole, cefotaxime, colistin, meropenem, and amikacin) was determined using the broth microdilution method according to the Clinical and Laboratory Standards Institute 2020 guidelines (CLSI M100-S30), with the exception of the break-point for tigecycline. The resistance break-point of tigecycline was interpreted as >2 mg/L according to the European Committee on Antimicrobial Susceptibility Testing guidelines (EUCAST, version 14.0) (https://www.eucast.org/clinical_breakpoints) (accessed on December 15^th^ 2022). The quality control strain was *E. coli* ATCC 25922.

### 2.3 Whole-genome sequencing and bioinformatics analysis

The genomic DNA of *tet*(X4)-positive isolates was extracted using a TIANamp Bacteria DNA Kit (Tiangen Biotech, China), and DNA libraries of these isolates were constructed using Illumina HiSeq 2500 (Annoroad Genomics Co.) and nanopore sequence platforms. The draft genome sequences of *E. coli* positive for *tet*(X4) were assembled using Unicycler (https://github.com/rrwick/Unicycler) (accessed on January 10^th^, 2023). The assembled genome sequences were annotated using PATRIC3.6.9 (https://patricbrc.org/) (accessed on January 11^th^, 2023). Sequence types (ST), AMR genes, and plasmid replicon types were identified using the CGE server (https://cge.cbs.dtu.dk/services/) (accessed on January 11^th^, 2023). Easyfig v2.2.3 (http://mjsull.github.io/Easyfig) (accessed on January 12^th^, 2023) was used to generate linear comparison figures and visualize the comparative genetic characteristics.

### 2.4 Conjugation assay

A conjugation experiment was performed to verify the transferability of *tet*(X4) in a *tet*(X4)-positive isolate (Yang et al., 2023) using streptomycin-resistant *E. coli* C600 as a recipient and the *tet*(X4)-positive strain as the donor. The donor and recipient bacteria were mixed in LB broth in a 1:3 ratio, and the mixture was transferred to a fresh LB agar plate with 0.22-μm sterile filter paper, followed by incubation at 37°C for 16 h. The transconjugants were detected by culturing on Mueller–Hinton plates containing 1000 mg/L streptomycin and 2 mg/L tigecycline. Finally, the transconjugants were verified by performing PCR using ERIC primers (F: 5′-ATGTAAGCTCCTGGGGATTCAC-3′, R: 5′-AAGTAAGTGACTGGGGTGAGCG-3′) (Yang et al., 2023). The frequency of conjugation transfer was calculated as the ratio of the number of transconjugants to the total number of recipients.

## 3 Results and discussion

### 3.1 Bacterial isolates

A strain positive for *tet*(X4), *E. coli* e6cp, was isolated from a duck fecal sample, representing a total prevalence of *tet*(X4) positivity in fecal samples of 0.14% (1/677). Comparatively, a previous study that assessed the prevalence of *E. coli* positive for *tet*(X4) on intensive pig and chicken farms in Hunan Province (Yang et al., 2023) detected six positive strains at a positivity rate of 2.3% (6/257). *tet*(X4) has similarly been also detected on pig and chicken farms in Jiangsu (18.24%, 24/159) (Wang et al., 2023), Shandong (66.7%, 40/60) (He et al., 2019), and Shanghai (12.24%, 6/49) (Wang et al., 2022) provinces. In humans, the prevalence of *tet*(X4) has gradually increased from a detection rate of 10.1% (11/109) (Ding et al., 2020) to 18.8% (147/782) (Zeng et al., 2022).

The prevalence of *tet*(X4)-positive strains in this study is thus somewhat lower than that which has been reported in previous studies on chicken and pig farms in China. We suspect that this discrepancy in prevalence rates could be attributed to the adoption of standardized management practices throughout all the assessed duck farms, including hatching and finishing, along with enhancements in captive breeding environments. Furthermore, implementing antimicrobial management and adhering to veterinary guidelines may have contributed to reducing antimicrobial resistance (Coyne et al., 2019).

### 3.2 Drug resistance phenotype and genotype

The resistance phenotype of the isolate reported in this study was determined by performing antimicrobial susceptibility tests using the broth microdilution method, with the minimum inhibitory concentrations (MICs) determined and compared with the resistance break-point according to the CLSI 2020 guidelines. The *tet*(X4)-positive *E. coli* exhibited resistance to a range of antibiotics, namely, chloramphenicol (>512 mg/L), nalidixic (16 mg/L), florfenicol (256 mg/L), trimethoprim-sulfamethoxazole (>16 mg/L), and tigecycline (8 mg/L), but was sensitive to cefotaxime (<0.125 mg/L), colistin (0.125 mg/L), meropenem (0.03 mg/L), and amikacin (2 mg/L) (**Table 1**).

**Table 1:** Characterization of the *tet*(X4)-positive *Escherichia coli*.

Strain	Species	Source	Sequence type	Antimicrobial agents (mg/L) ^1^								
				TGC	CPL	NAL	FFC	STX	CTX	COL	MEM	AMK
e6cp	*E. coli*	Duck feces	ST10776	**8**	**> 512**	**> 16**	**256**	**> 16**	< 0.125	0.125	0.03	2

^1^ Abbreviations and antibiotic resistance break-points according to the Clinical and Laboratory Standards Institute (CLSI) 2020 guidelines: TGC: tigecycline (R > 2 mg/L), CPL, chloramphenicol (R > 8 mg/L); NAL, nalidixic acid (R > 4 mg/L); FFC, florfenicol (R > 16 mg/L); SXT, trimethoprim–sulfamethoxazole (R > 4 mg/L); CTX, cefotaxime (R > 2 mg/L); COL, colistin (R > 2 mg/L); MEM, meropenem (R > 8 mg/L;, AMK, amikacin (R >16 mg/L). Bold underlined numbers indicate resistance to the corresponding antimicrobial agents.

On the basis of whole-genome sequencing (WGS) and subsequent standalone BLAST analysis against ResFinder, we identified eight resistance genes coexisting in the isolated strain, namely, *tet*(X4), *floR*, *tet*(A), *bla*_TEM-1B_, *aph(6)-Id*, *dfrA*14, *qnrS1*, and *sul*3. Only aph(6)-Id, blaTEM-1B, qnrS1, and sul3 were identified in IncX1 plasmid. Notably the *tet*(X4) gene was carried within a separate circular. Most of these genes were consistent with the resistance phenotype, although there were some exceptions, For example, despite the presence of the resistance genes, *aph*(6)-Id and *bla*_TEM-1B_, the strain did not exhibit resistance to amikacin, cefotaxime, or meropenem, and conferred resistance only to older-generation antibiotics. In contrast, a high level of chloromycetin resistance (>512 mg/L) was observed in the absence of any corresponding resistance genes.

### 3.3 Genetic environments and transferability of *tet*(X4)

**Figure 1:**
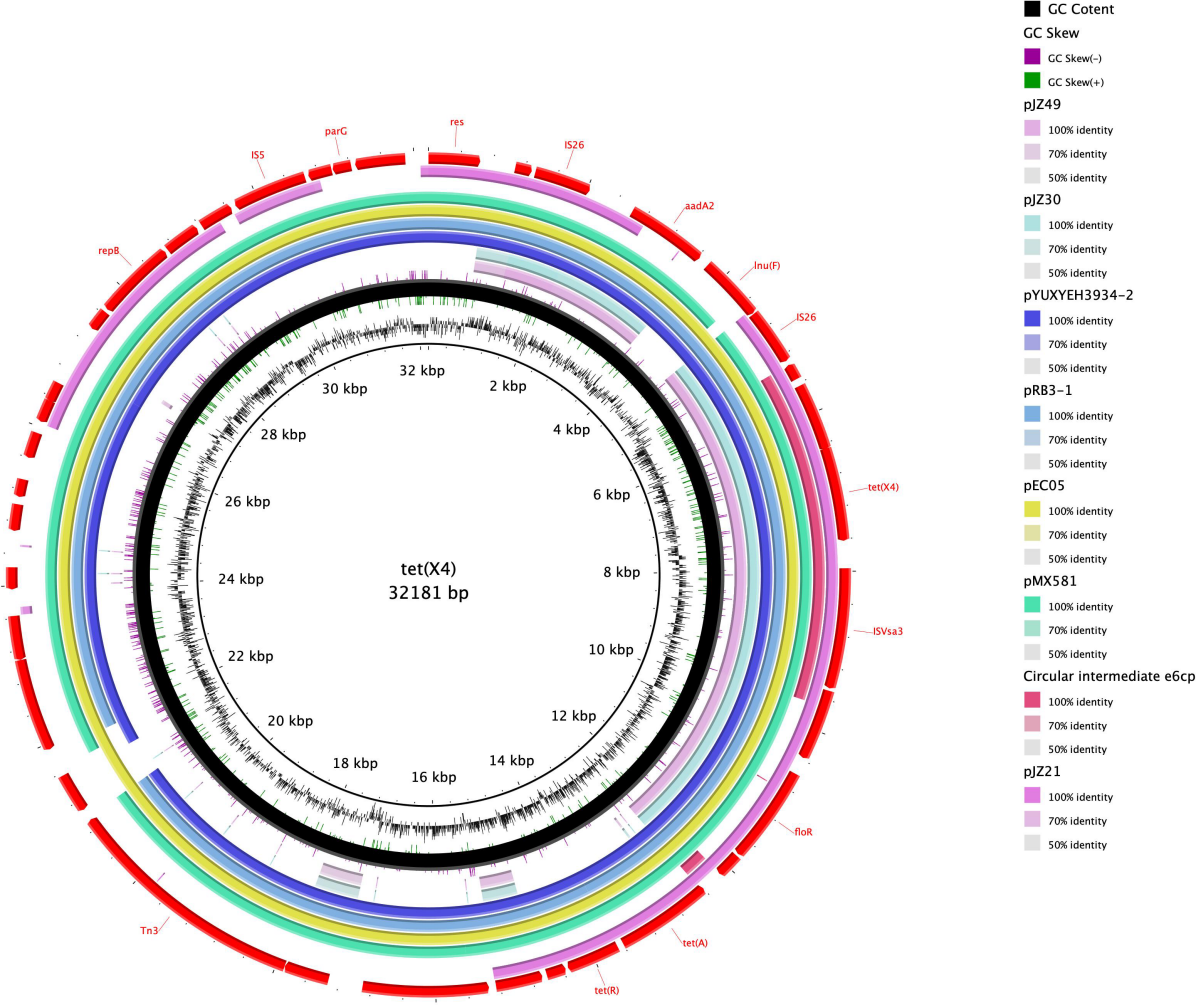
Circular comparison of the *tet*(X4)-bearing Circular intermediate e6cp with other similar plasmids obtained from the NCBI database. The outermost ring indicates the reference plasmid with the respective gene positions. The *tet*(X4)-bearing Circular intermediate e6cp is shown in pink.

**Figure 2:**
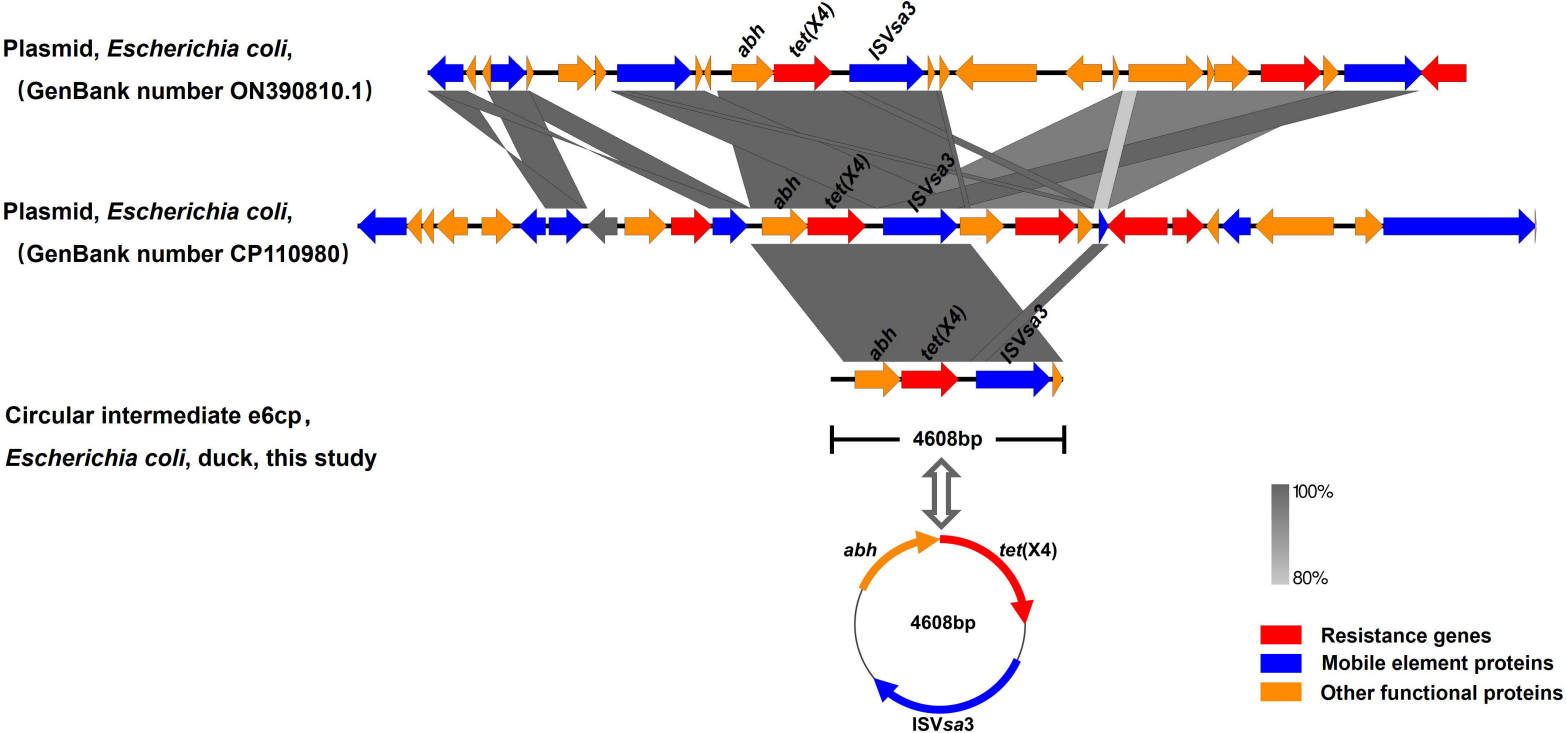
Comparison of the genetic context of *tet*(X4) with those of closely-related sequences. The position and orientation of genes are indicated by arrows and labeled with gene names. Resistance genes, mobile element proteins, and other functional proteins are indicated by red, blue, and orange arrows, respectively.

The genome sequence of the *tet*(X4)-positive strain was determined based on WGS, and bioinformatic analysis revealed that the strain is characterized by a multidrug resistance gene phenotype and belongs to a new type of sequence, ST10776. This discovery thus provides evidence to indicate that the spread of *tet*(X4) is not attributable to clonal dissemination (**Table 1**). Splicing results indicated that *tet*(X4) is located within a circular intermediate (4608 bp). The sequence upstream of *tet*(X4) contains an alpha/beta hydrase (*abh*) and an IS*Vsa3* (mobile element protein), which form the genetic environment of the IS*Vsa3*-ORF2-*abh*-*tet*(X4) circular intermediate (**Figures 1** and **2**). It was highly consistent with p34AB plasmid (170312-bp) from *E.coli* in 2019 (He et al., 2019), which contains a highly mobile 5586-bp region “IS*Vsa3*-ORF2-*abh*-*tet*(X4)-IS*Vsa3*”. Reverse PCR has demonstrated that the region “IS*Vsa3*-ORF2-*abh*-*tet*(X4)-IS*Vsa3*” could form a circular intermediate “IS*Vsa3*-ORF2-*abh*-*tet*(X4)”, which insert to other IS*Vsa3* positive plasmid and mediated transfer (He et al., 2019). In this study, the existence of the circular intermediate “IS*Vsa3*-ORF2-*abh*-*tet*(X4)” was directly discovered for the first time through Whole-genome sequencing.

Upon comparison of the genetic environment, the backbone of the circular intermediate e6cp identified in the present study was found to exhibit 100% coverage and 99% identity with pJZ49-*tet*(X4) (GenBank accession number ON390810) in *Proteus mirabilis* isolated from swine and 94% coverage and 99% identity with pYUXYEH3934-2 (GenBank accession number CP110980) in *E. coli* isolated from *Homo sapiens* (**Figure 2**). Moreover, the circular intermediate e6cp.showed 100% identity and 6.3% coverage with pJZ21 (GenBank accession number ON390807.1), which was isolated from swine (**Figure 1**). The circular intermediate, IS*Vsa3*-ORF2-*abh*-*tet*(X4) identified by WGS in the present study would tend to indicate that *tet*(X4) will continue to spread and pose a potential risk to public health. Furthermore, similar genetic contexts in diverse sources and strains are conducive to the ongoing spread of this resistance gene.

IS*Vsa3* is an important mobile element that mediates the horizontal translocation of *tet*(X4) between plasmids (Aminov, 2021; Li et al., 2021; Wang et al., 2022). To investigate whether IS*Vsa3* could mediate *tet*(X4) gene transmissibility, we subjected the positive strain to conjugation experiments with *E. coli* C600, and accordingly found that the strain successfully transferred *tet*(X4) to *E. coli* C600 at a frequency of 10^-5^. The result of the antimicrobial susceptibility test revealed that the MIC values of these transconjugants increased from 0.5 mg/L to 4 mg/L, thereby conferring resistance to tigecycline. These results imply that the *tet*(X4) gene of the positive strain is harbored within a conjugative plasmid, the transmission of which is mediated by IS*Vsa3* elements. At 4608 bp in size, this circular intermediate is relatively and simple in structure, and is characterized by enhanced mobility and transferability, thereby indicating that the horizontal dissemination of *tet*(X4) via conjugative circular intermediate or other mobilizable genetic elements may have occurred on the duck farm from which the *E. coli* e6cp strain was isolated.

In summary, to the best of our knowledge, this is the first study in which the *tet*(X4) tigecycline resistance gene has been detected on a duck farm in Hunan Province. Our investigation of the prevalence and characteristics of *tet*(X4) indicated that this gene was detected exclusively in duck fecal samples at a positivity rate of 0.14% (1/677), which is lower than that previously reported. Furthermore, *tet*(X4) was established to be harbored a circular intermediate (4608 bp) within the genetic environment of the IS*Vsa3*-ORF2-*abh*-*tet*(X4) elements and could be transferred to other chromosomes and plasmids. Compared with previously reported *tet*(X4)-carrying plasmids, we found that this circular intermediate was inserted into different bacterial sequence types and conferred high-level resistance to tigecycline. Considering the management model of duck farms, each farm maintains fixed breeders that do not intermingle with others, and the use of medications on these farms adheres strictly to veterinarian-prescribed protocols. We hypothesize that (1) the establishment of informational supervision throughout the industry chain is conducive to the prevention and control of antimicrobial resistance, and (2) horizontal transmission plays an important role in the spread of the *tet*(X4) gene and is a potential transmission route that facilitates the spread of antimicrobial resistance genes. However, the number of positive *tet*(X4) samples in this study was insufficiently large to clarify the transmission mechanism. Moreover, the circular intermediate of *tet*(X4) identified in study is highly mobile and transferable, thereby highlighting the threat posed by the spread of genes with resistance to tigecycline within this duck farm, and potentially other farms. Consequently, it is imperative to strengthen the scientific use of antibiotics in poultry farm and conduct daily monitoring of tigecycline resistance genes.

## Data Availability

The datasets presented in this study can be found in online repositories. The names of the repository/repositories and accession number(s) can be found in the article/supplementary material.
